# Correlation between oxidative stress and inflammation with metabolomics profile in skeletal muscle of ageing animal model and its modulation by tocotrienol-rich fraction

**DOI:** 10.3389/bjbs.2026.16208

**Published:** 2026-05-08

**Authors:** Siti Liyana Saud Gany, Nur Fatin Nabilah Mohd Sahardi, Jen Kit Tan, Suzana Makpol

**Affiliations:** 1 Department of Biochemistry, Faculty of Medicine, Universiti Kebangsaan Malaysia, Kuala Lumpur, Malaysia; 2 Secretariat of Research & Innovation, Faculty of Medicine, Universiti Kebangsaan Malaysia, Kuala Lumpur, Malaysia; 3 Ageing and Degenerative Diseases Research Group, Universiti Kebangsaan Malaysia, Bangi, Selangor, Malaysia

**Keywords:** ageing, DNA damage, low-grade inflammation, oxidative stress, sarcopenia

## Abstract

**Introduction:**

Sarcopenia, characterised by age-associated decline in skeletal muscle mass and function, is driven by multifactorial mechanisms including oxidative stress, chronic inflammation, and genomic instability. The imbalance between reactive oxygen species (ROS) and antioxidant defence contributes to mitochondrial dysfunction and DNA damage, thereby affecting cellular metabolism and promoting muscle degeneration. Tocotrienol-rich fraction (TRF), a potent antioxidant form of vitamin E, has shown potential in modulating oxidative and inflammatory pathways. However, *in vivo* evidence exploring TRF’s multifaceted role in ageing muscle remains limited. This study investigates the correlation between oxidative stress and inflammation with the metabolomics profile in ageing skeletal muscle in a rat model and its modulation by tocotrienols.

**Methods:**

Young (3 months) and old (21 months) male Sprague-Dawley rats were divided into control and TRF-supplemented groups (n = 10). TRF was administered orally (60 mg/kg/day) for 3 months. Antioxidant enzymes, lipid peroxidation products: malondialdehyde (MDA) and 4-hydroxynonenal (4-HNE); and inflammatory markers: C-reactive protein (CRP), Interleukin-6 (IL-6), and tumour necrosis factor alpha (TNF-α) were quantified, alongside DNA damage, using the comet assay. Muscle histology was assessed using hematoxylin and eosin (H&E) staining. Pearson correlation analysis was performed between selected metabolites and biological markers.

**Results:**

Ageing significantly increased oxidative damage, pro-inflammatory markers, and DNA fragmentation, while reducing antioxidant enzyme activities and disrupting metabolic profiles. Although TRF supplementation did not significantly restore muscle mass or overall body composition, it effectively enhanced antioxidant defence by increasing Superoxide dismutase (SOD) and catalase (CAT) activities, reducing lipid peroxidation (MDA and 4-HNE), attenuating inflammatory responses, preserving DNA integrity, and improving muscle histological features. Importantly, correlation analyses revealed that ageing is associated with a coordinated metabolic shift linking amino acid and carnitine metabolism with antioxidant defence, inflammation, and genomic stability. TRF supplementation weakened these maladaptive biomarker and metabolite associations while strengthening correlations between protective metabolites (e.g., taurine, histidine, pantothenic acid) and antioxidant enzymes, alongside inverse relationships between lipid peroxidation and inflammatory markers (e.g., MDA, prostaglandin factor 2-alpha, PGF2α) and redox-supportive metabolites.

**Discussion:**

Collectively, these findings indicate that TRF acts primarily as a preventive intervention by restoring redox balance, dampening inflammatory signalling, and stabilising metabolic and inflammatory coupling, highlighting its potential as a nutritional strategy for preserving muscle health and genomic integrity during ageing.

## Introduction

As individuals age, they often experience a steady loss of skeletal mass, strength, and functional ability, a condition known as sarcopenia [1]. While some degree of muscle loss is a normal aspect of ageing, sarcopenia represents a more severe, clinically relevant decline that adversely affects mobility, contributes to physical impairment, and diminishes overall quality of life [2, 3]. Interestingly, the prevalence of sarcopenia varies considerably across populations and age groups, with estimates indicating that 5%–13% of individuals aged 60–70 years old are affected, increasing dramatically to 11%–50% in those aged 80 years old and above [4, 5]. This rising prevalence highlights the urgent need for regular screening and early intervention, particularly among high-risk groups such as older adults and individuals with comorbid conditions like cancer or chronic kidney disease [6, 7].

Ageing is accompanied by an increase in oxidative stress, which arises when the production of ROS surpasses the capacity of the body’s antioxidant defense. The increase in ROS contributes to cellular damage, particularly within skeletal muscle tissue [8, 9]. In addition, the increased oxidative burden impairs mitochondrial function, heightening the risk of muscle atrophy and facilitating the progression of sarcopenia [10, 11]. Notably, age-related ROS accumulation can activate apoptotic signalling pathways, leading to muscle cell death and a consequent decline in both muscle mass and function [12]. Furthermore, oxidative damage to mitochondrial DNA (mtDNA) may worsen these effects by disrupting mitochondrial efficiency, thereby perpetuating a feedback loop of declining mitochondrial function and increasing oxidative stress [13, 14].

Inflammation is a significant contributor to the ageing process and is commonly referred to as “inflammaging”, a condition characterised by chronic, low-grade inflammation [15]. Increased concentrations of proinflammatory cytokines, including tumour necrosis factor alpha (TNF-α) and interleukin (IL-6), have been closely linked to the loss of muscle mass and reduced muscle strength [16, 17]. Inflammation also disrupts muscle protein synthesis, leading to impaired regeneration and loss of muscle mass [18]. The close relationship between oxidative stress and inflammation underscores the potential of anti-inflammatory strategies to prevent or slow the progression of sarcopenia.

DNA damage is a key contributor to both ageing and sarcopenia. In muscle cells, the accumulation of DNA lesions has been associated with increased senescence and apoptosis, ultimately leading to muscle loss [19]. Previous studies have shown that aged muscle cells are more prone to damage compared to younger ones [20–22]. This genomic instability compromises the regenerative capacity of ageing muscles, further accelerating functional decline [23].

Current management strategies typically emphasize resistance training and targeted nutritional support to enhance muscle mass and function, underscoring the condition’s modifiability through lifestyle intervention [24, 25]. Recognized as a geriatric syndrome, the timely diagnosis and treatment of sarcopenia are critical for improving patient outcomes and mitigating risks of adverse events, including falls, disability, and mortality [26, 27].

The potential of tocotrienol-rich fraction (TRF), a subgroup of the vitamin E family, as a therapeutic strategy against ageing and sarcopenia is gaining increasing attention. Structurally, tocotrienols possess an unsaturated isoprenoid side chain, which enhances their mobility within lipid bilayers and facilitates more efficient incorporation into cell membranes, particularly in metabolically active tissues such as skeletal muscle [28]. This structural distinction contributes to greater antioxidant efficiency, particularly through enhanced incorporation into lipid bilayers, enabling more effective protection of mitochondrial and cellular membranes against lipid peroxidation [29]. This antioxidant effect is essential, as an overabundance of free radicals can harm skeletal muscle cells, leading to reduced muscle function and cellular ageing. Importantly, TRF has been shown to enhance the expression of essential antioxidant enzymes, such as superoxide dismutase and glutathione peroxidase, which play a vital role in safeguarding cells against oxidative damage [29]. In addition to direct free radical scavenging, TRF also exerts non-antioxidant regulatory effects, including the modulation of antioxidant enzyme expression such as SOD and CAT, as well as the activation of redox-sensitive signalling pathways, notably nuclear factor erythroid 2-related factor 2 (Nrf2), which play critical roles in maintaining cellular redox homeostasis during ageing [29, 30].

In addition, tocotrienols have been shown to regulate proinflammatory cytokine levels, thereby reducing the inflammatory environment that contributes to muscle degeneration [31]. Recent preclinical research indicates that TRF supplementation may help maintain mitochondrial function, minimize oxidative damage in muscle tissue, and enhance muscle fibre regeneration, positioning it as a promising option for addressing sarcopenia [32–34]. However, despite these promising findings, evidence from *in vivo* models remains limited, particularly in the context of integrated analysis of oxidative stress, inflammation, and DNA damage in ageing. Thus, further investigation is warranted to elucidate the mechanistic effects of TRF on muscle health during ageing and to explore its potential as a multifaceted intervention against sarcopenia.

While previous studies have primarily examined oxidative stress, inflammation, or metabolic alterations in isolation, ageing is increasingly recognized as a condition characterized by dysregulation of interconnected biological networks. Therefore, the present study adopts a metabolite-biomarker correlation approach to provide an understanding of how metabolic pathways interact with oxidative stress, inflammation, and DNA damage. This approach allows for the identification of network-level interactions rather than isolated endpoints, which is particularly relevant in complex, multifactorial conditions such as ageing and sarcopenia. Importantly, this integrative analysis enables evaluation of how TRF modulates the coupling between metabolic and physiological processes, representing a key strength and novel contribution of the present study.

## Materials and methods

### Animal groups and experimental design

Forty male Sprague-Dawley (SD) rats, aged 3 months, were purchased from the Laboratory Animal Resources Unit (LARU), Universiti Kebangsaan Malaysia. The animals were randomly assigned into two age groups: young (3 months old) and old (21 months old). Only male rats were used in this study to minimize variability associated with hormonal fluctuations, particularly those related to the estrous cycle, which can influence oxidative stress, inflammatory responses, and metabolic profiles [35]. This approach is commonly adopted in ageing and metabolomics studies to improve internal consistency and reduce biological variability, thereby allowing clearer interpretation of intervention effects. Each age group was randomly subdivided into control and supplemented subgroups, comprising 10 rats per subgroup, based on a previous study, with slight modifications [36]. All animals were assumed to be healthy at the time. Following a one-week acclimatisation period, rats were individually housed with free access to food and water *ad libitum*. The control groups received refined, bleached, and deodorized (RBD) palm olein. In contrast, the supplemented groups were administered a tocotrienol-rich fraction (TRF) at 60 mg/kg body weight/day over 3 months via oral gavage [37]. Blood samples were collected via retro-orbital sinus at baseline and at 1.5 and 3 months following the start of supplementation. Upon completion of the supplementation period, all animals were euthanized, and the gastrocnemius soleus muscles from both hind limbs were excised, rapidly frozen in liquid nitrogen, and stored at −80 °C for subsequent analysis. All experimental procedures were conducted in accordance with institutional ethical guidelines and were approved by the Universiti Kebangsaan Malaysia ethical committee (approval number BI-OK/FP/2020/SUZANA/25-MAR/1099-MAR-2020DEC.2022). Group allocation, experiment conduct, outcome assessment, and data analysis were all performed by investigators with knowledge of the group assignments (no blinding applied).

### Preparation of TRF supplementation

TRF (Golden TriTM E70) and RBD palm olein were generously supplied by SIME Darby Plantation Berhad (Selangor, Malaysia). Each Gram of Gold TriTM E70 contained 74% tocotrienols, comprising 24% α-tocotrienol, 4% β-tocotrienol, 32% γ-tocotrienol, and 14% δ-tocotrienol. The remaining content consisted of alpha-tocopherol. The RBD palm olein was characterised by 0.054% free fatty acids, 0.043% moisture and impurities, 0.45% peroxide values, and a 64.71% iodine value. The TRF supplementation was prepared weekly by mixing 2.4 g of TRF with 40 mL of RBD palm olein in a Falcon tube under light-protected conditions. The mixture was vortexed thoroughly to ensure homogeneity, wrapped in aluminum foil, and stored at 4 °C until use.

### Measurement of bone integrity via DEXA

Bone integrity was assessed using dual-energy X-ray absorptiometry (DEXA) to determine bone mineral content (BMC), bone mineral density (BMD), fat percentage, fat mass, lean mass + bone mineral content, and total mass. Whole-body DEXA scan utilizes two low-dose X-ray beams that are differentially absorbed by bone and soft tissue, enabling differentiation among fat, bone mineral, and lean tissue based on their density profile. During scanning, a movable arm passed over the rat’s body to measure bone density along the central skeletal axis. Each scan was non-invasive and completed within approximately 4 min.

### Measurement of oxidative stress and inflammatory markers

Plasma levels of interleukin-6 (IL-6) and tumour necrosis factor-alpha (TNF-α) were measured using the PrimePlex Multiplex Protein Detection assay service provided by Prima Nexus Sdn Bhd (Selangor, Malaysia), a multiplex platform that enables simultaneous analysis of multiple biomarkers within a single panel. Plasma C-reactive protein (CRP) levels were measured using an ELISA kit (Cat No: ELK1055; ELK Bio-technology Co. Ltd., Wuhan, China). Serum levels of superoxide dismutase-1 (SOD-1; Cat No: ELK8178), lipid peroxide (LPO; Cat No: ELK8896), and catalase (CAT; Cat No: ELK5986) were measured using ELISA kits from ELK Biotechnology Co. Ltd. Each serum sample was analysed in duplicate. Levels of malondialdehyde (MDA; Cat No: ELK8612) and 4-hydroxynonenal (HNE; Cat No: ELK8373) in each muscle tissue were measured in triplicate using ELISA kits from ELK Biotechnology Co. Ltd. Each gastrocnemius muscle tissue sample was tested in duplicate. This study was designed as a hypothesis-testing experiment with oxidative stress markers (SOD, CAT, MDA, 4-HNE) and inflammatory markers (CRP, IL-6, TNF-α, PGF-2α) defined as the primary outcome measures used to guide sample size determination.

### Assessment of DNA damage by the comet assay

The comet assay was performed with slight modifications to the protocol described by Singh et al. [38]. Fully frosted microscope slides were initially coated with a thin layer of 0.6% normal melting point agarose (Sigma-Aldrich, St. Louise, MO, USA), followed by the placement of a coverslip to ensure even spreading. The slides were then left at room temperature for 10 min to allow the gel to solidify. A second layer was then added by mixing 5 μL of whole blood with 0.6% low-melting-point agarose. The slides were placed on ice for 20 min to ensure complete solidification. Following this, coverslips were gently removed, and the slides were immersed in freshly prepared, cold lysing solution (2.5 M NaCl, 100 mM Na_2_EDTA-2H_2_O, 10 mM Tris at pH 10, 1% sodium N-lauroylsarcocinate, 1% Triton X-100, and 10% dimethyl sulfoxide) for one hour at 4 °C. Subsequently, the slides were transferred to a horizontal electrophoresis tank filled with freshly prepared cold buffer (0.3 M NaOH and 1 mM Na2EDTA) and incubated for 20 min to allow DNA unwinding. Electrophoresis was then carried out for 20 min at 25 V, with the current adjusted to 300 mA. After electrophoresis, the slides were rinsed three times with neutralization buffer (0.4 M Tris Base, pH 7.5) to remove residual alkali, then stained with ethidium bromide (20 μg/mL) (Sigma-Aldrich). The slides were air-dried at room temperature in preparation for analysis. DNA migration patterns were observed under a fluorescence microscope (Olympus BX53, Shinjuku, Tokyo, Japan) at ×40 magnification. Photomicrographs of 500 randomly selected, nonoverlapping cells per slide were captured. Each sample was prepared in duplicate. All procedures were conducted under low-light conditions to minimize artificial DNA damage and to avoid fluorescent dye bleaching. Comet images were analysed using ImageJ software (National Institutes of Health and the Laboratory for Optical and Computational Instrumentation (LOCI, University of Wisconsin)). Parameters used to assess DNA damage included tail DNA percentage, tail length, tail moment, and olive moment.

### Histological analysis

Gastrocnemius and soleus muscle tissue were fixed in 10% neutral buffered formalin to ensure long-term preservation. The gastrocnemius muscle tissue was sectioned longitudinally, while the soleus muscle tissue was sectioned transversely. Both tissue types were placed in plastic cassettes processed using an automated vacuum tissue processor (STP 120 Spin Tissue Processor, Thermo Fisher Scientific, Waltham, Massachusetts, USA). Following fixation and processing, the tissue was embedded in paraffin blocks (Surgipath® Paraplast®, Leica Biosystems Richmond Inc., Illinois, USA). Tissue sections were cut at a thickness of 5 μM using a manual rotatory microtome (RM2135 Manual Rotatory Microtome, Leica, Nussloch, Germany). These sections were floated in a 38 °C water bath (Leica HI1210 Water Bath, Leica, Nussloch, Germany) to flatten them before mounting on a glass microscope slide. The mounted slides were subsequently dried in a 50 °C oven for 30 min (Menmert, Buechenbach, Germany). Hematoxylin and eosin (H&E) staining was performed using an automated slide stainer (Leica, Biosystem, Deer Park, Illinois, USA). After staining, slides were mounted with DPX mounting medium (VWR International Ltd., Poole, England) and coverslips were applied. Stained sections were examined under a light microscope (Olympus BX53, Tokyo, Japan), and representative images were captured for analysis.

### Metabolomics analysis

The metabolomics analysis used in this study was derived from our previously published work Saud Gany et al. [39], which employed an untargeted liquid chromatography–mass spectrometry (LC-MS)-based platform for muscle tissue metabolite profiling. Briefly, gastrocnemius muscle samples were extracted and analysed using LC-MS, followed by data preprocessing including peak detection, alignment, and normalisation. Metabolite identification was performed based on database matching and MS/MS spectral confirmation.

Data were subjected to appropriate scaling and statistical analysis, including multiple testing correction, to identify significantly altered metabolites. Full details of the analytical platform, sample preparation, quality control procedures, metabolite identification criteria, and statistical thresholds are described in the original publication. In the present study, these previously identified metabolites were used for correlation analyses with oxidative stress, inflammatory, and DNA damage markers.

### Statistical analysis

Statistical analysis was performed using GraphPad Prism 10.0 Software. Data were first tested for normality using the Shapiro-Wilk test. For data that followed a normal distribution, a two-way ANOVA was conducted, followed by Tukey post-hoc tests. All results are presented as mean ± standard deviation (SD), with a significant value of p < 0.05. Pearson’s correlation analysis was performed between oxidative stress markers, anti-inflammatory markers, and DNA damage parameters, with differentially expressed metabolites identified in a previous metabolomics study [39].

## Results

### Effect of TRF on bone integrity

**FIGURE 1:**
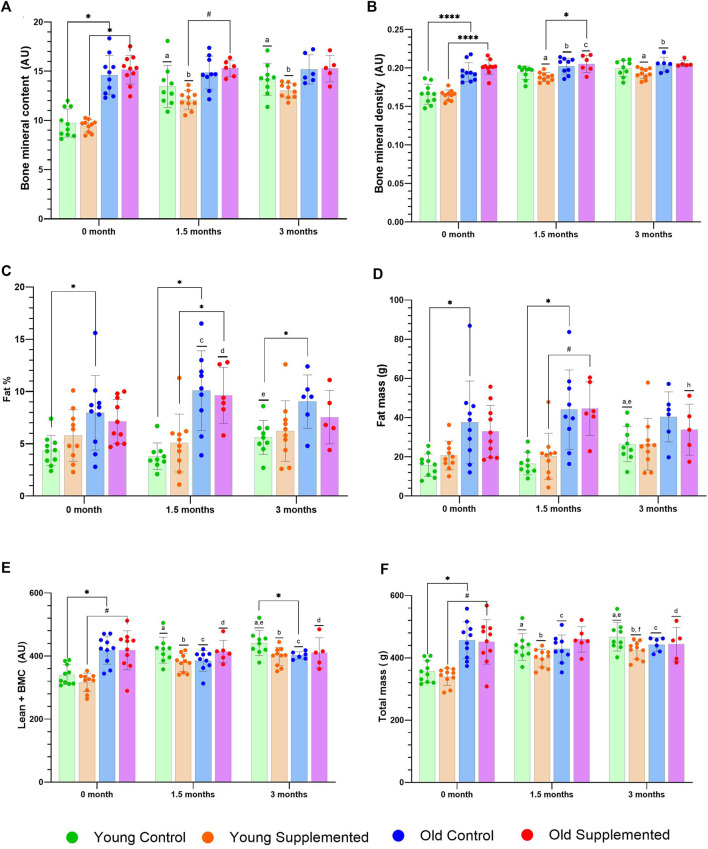
Bone integrity status following TRF supplementation. Bone mineral content **(A)**, bone mineral density **(B)**, fat percentage **(C)**, fat mass **(D)**, lean mass + bone mineral content **(E)**, and total mass **(F)** for the control and supplementation groups in young and aged rats. Symbols indicate significant differences at p < 0.05; a significant difference compared to the young control group at 0 month; b significant difference compared to the young supplemented group at 0 month; c significant difference compared to the old control group at 0 month; d significant difference compared the old supplemented group at 0 month; e significant difference compared to the young control group at 1.5 months; f significant difference compared to the young supplemented group at 1.5 months; h significant difference compared to the old supplemented group at 1.5 months; * significant difference compared to the control group at the same time point; and # significant difference compared to the supplemented group at the same time point. Statistical analysis was performed using ANOVA followed by Tukey’s *post hoc* test.

Figure 1 depicts the assessment of bone mineral content (BMC), bone mineral density (BMD), fat percentage, fat mass, lean mass combined with BMC, and overall mass through DEXA scanning. Analysis of BMC revealed a significantly higher level at 0 month in the old control and old supplemented groups compared to their young counterparts (Figure 1A). Additionally, both the young control and supplemented groups showed significant increases at 1.5 and 3 months compared to their baseline. Similar trends were observed in BMD analysis, with the old control and old supplemented groups demonstrating significantly higher levels than their younger counterparts (Figure 1B). Furthermore, both the young control and supplemented groups showed a significant increase at 1.5 and 3 months of TRF supplementation compared with baseline.

At 1.5 months, the old control group had a significantly higher fat percentage than the young control group (Figure 1C). Similarly, the fat percentage in the old supplemented group increased significantly from baseline. Both the young control and supplemented groups also showed notable increases in fat percentage relative to their respective baseline measurements at 0 month. These patterns were consistent with the observed changes in fat mass (Figure 1D). Additionally, when evaluating lean mass in combination with bone mineral content (BMC), both the old control and old supplemented groups showed significantly higher values than those in the younger groups (Figure 1E). Both the young control and supplemented groups showed significant increases after 1.5 and 3 months compared to their baseline at 0 month, while the old control group exhibited a significant decrease in lean mass combined with BMC at 1.5 months compared to its baseline. For total mass, the old control and supplemented groups showed significantly higher values than the young control and supplemented groups (Figure 1F). Additionally, both the young control and supplemented groups exhibited significant increases after 1.5 and 3 months compared to baseline at 0 month. These changes showed that ageing alters body composition; however, supplementation with TRF in this study did not significantly reverse these effects.

### TRF reduces oxidative stress in the blood and muscle

**FIGURE 2:**
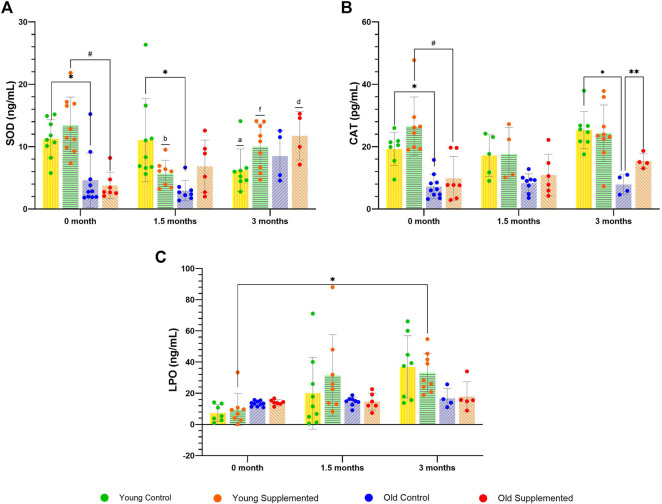
Levels of oxidative stress activities for the young control group, young supplemented group, old control group, and old supplemented group at three different time points: 0 month, 1.5 months, and 3 months of TRF supplementation. **(A)** Superoxide dismutase, **(B)** Catalase activity, and **(C)** Lipid peroxidation. Symbols denote significant values, p < 0.05. * Indicates significant difference between young supplemented and young control group; # indicates significant difference between old supplemented and old control group; ** indicates significant difference between control and supplemented group; b indicates significant difference with the young supplemented group at 0 month; f indicates significant difference with the young supplemented group at 1.5 months; and d indicates significant difference with the old supplemented group at 0 month. Data are presented as mean ± SD, and analysed using RM ANOVA with Tukey’s *post hoc* analysis. N = 34 (n = 5-8, young control group; n = 4-9, young supplemented group; n = 4–10, old control group; and n = 4-7, old supplemented group.

The investigation into oxidative stress markers yielded several noteworthy findings. Firstly, significant changes were observed in superoxide dismutase (SOD) activity: the young control group showed a notable decrease after 3 months compared to baseline and 1.5 months, whereas the young supplemented group showed a significant increase after 3 months (Figure 2A). Moreover, the old supplementation group showed a significant increase in SOD activity after 3 months. Secondly, catalase (CAT) activity varied significantly among experimental groups, with the old control group showing a significant reduction compared to the young control group, both at baseline, and its level increased in older rats after 3 months of TRF supplementation (Figure 2B). However, the old supplemented group noted an intriguing increase in CAT activity after 3 months compared to their respective controls. Furthermore, lipid peroxidation (LPO) levels increased significantly in both the control group and the young supplemented group after 3 months, compared to baseline, and in the old control group compared to the young control group at baseline (Figure 2C).

**FIGURE 3:**
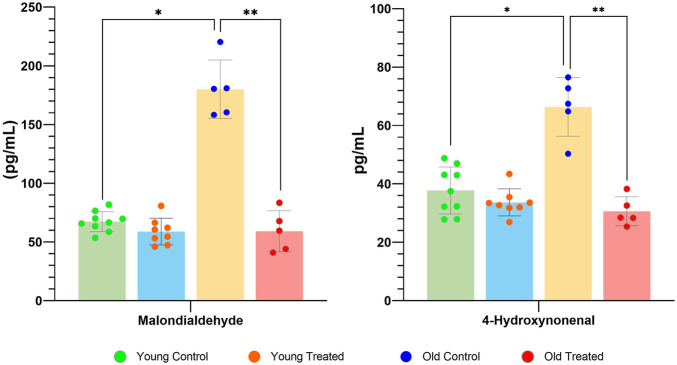
Levels of Malondialdehyde (MDA) and 4-Hydroxynoneal (HNE) for the young control group, young supplemented group, old control group, and old supplemented group. Symbols denote significant values, p < 0.05. * Indicates significant difference between the young control group and old control group, ** indicates significant difference between the old control group and old supplemented group. Data are presented as mean ± SD, and analysed using ANOVA with Tukey’s *post hoc* analysis. N = 27 (n = 9, young control group; n = 5, old control group; n = 5, old supplemented group; n = 5, old supplemented group.

Finally, both malondialdehyde (MDA) and 4-hydroxynonenal (4-HNE) levels demonstrated a significant increase int he old control group compared to the young control group (Figure 3). Conversely, a significant decrease in MDA and 4-HNE levels was observed in the old supplementation group compared with their respective controls. These findings underscore the intricate relationship between oxidative stress markers in ageing rats and highlight the potential benefits of TRF supplementation in attenuating oxidative damage.

### TRF suppresses inflammation in the blood

**FIGURE 4:**
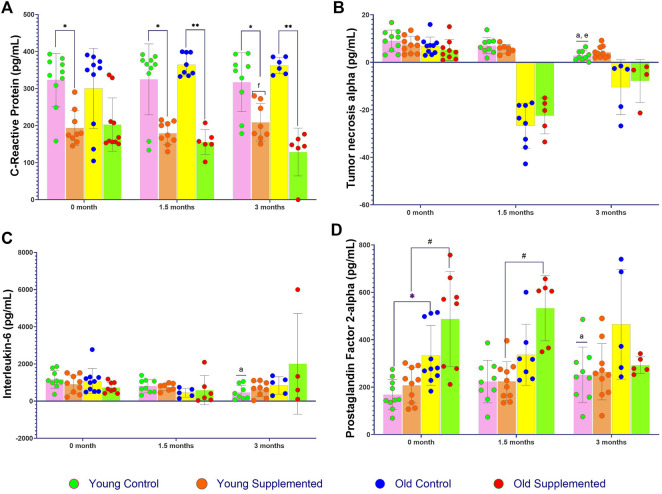
Levels of inflammatory markers of the young control group, young supplemented group, old control group, and old supplemented group at three different time points: 0 month, 1.5 months, and 3 months. **(A)** C-Reactive protein; **(B)** Tumour necrosis alpha; **(C)** Interleukin-6; and **(D)** Prostaglandin Factor 2-alpha. Symbols denote significant values, p < 0.05. * Indicates significant difference between the young control group and the young supplemented group; ** indicates significant difference between the old control group and the old supplemented group; a indicates significant difference with the young control group at 0 month; e indicates significant difference with the young control group at 1.5 months; and f indicates significant difference with the young supplemented group at 1.5 months. Data are presented as mean ± SD, and analysed using ANOVA with Tukey’s *post hoc* analysis. N = 39 (n = 9–10, young control group; n = 9, young supplemented group; n = 5–10, old control group; n = 4–10, young supplemented group.

Analysis of CRP levels revealed a significant difference in the young supplemented group at 3 months compared to the same group at 1.5 months (Figure 4A). Additionally, significantly lower CRP levels were observed in the old supplemented group compared to the young supplemented group at 3 months. Evaluation of TNF-α levels demonstrated a statistically significant reduction in the young control group at 3 months compared to 0 month. Overall, after 1.5 months of TRF supplementation and continuing through to 3 months, TNF-α concentrations in both the old control and old supplemented groups declined to below detectable levels (Figure 4B). Analysis of IL-6 levels showed a significant difference in the young control group, particularly at 3 months after TRF supplementation compared to baseline (0 month) (Figure 4C). No significant differences in IL-6 levels were observed across the experimental time points in other groups.

PGF-2α levels, which were measured in the rat urine, showed significantly higher levels in the old control rats compared to the young control group at 0 month (Figure 4D). Similarly, the old supplemented group exhibited significantly higher PGF-2α levels than the young supplemented group at 0 month. After 1.5 months, a significant increase in PGF-2α was observed in the old supplemented group compared to the young supplemented group. Furthermore, at 3 months, the young control group showed a significant increase in PGF-2α levels compared to its baseline value at 0 month.

### TRF affects DNA integrity in whole blood

**FIGURE 5:**
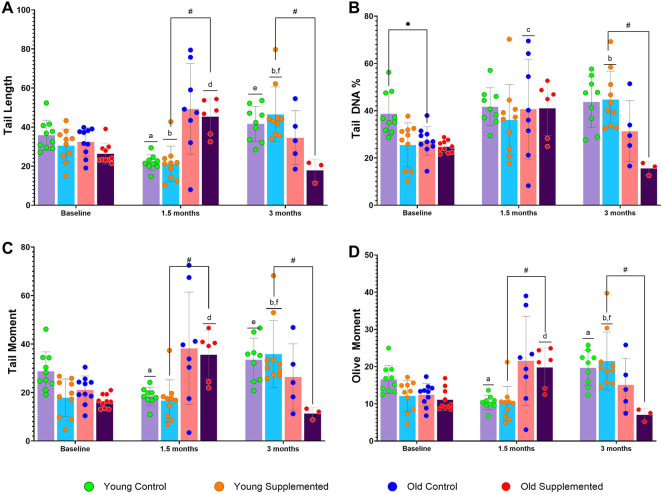
DNA damage levels of the young control group, young supplemented group, old control group, and old supplemented group at three different time points: 0 month, 1.5 months, and 3 months. **(A)** Tail length, **(B)** Tail DNA%, **(C)** Tail moment, and **(D)** Olive moment. Symbols denote significant values, p < 0.05. * indicates significant difference between young control group and old control group; # indicates significant difference between young supplemented group and old supplemented group; a indicate significant difference compared to young control group at 0 month; b indicates significant difference compared to young supplemented group at 0 month; c indicates significant difference compared to old control group at 0 month; d indicates significant difference compared to old supplemented group at 0 month; e indicates significant difference compared to young control group at 1.5 months; and f indicates significant difference compared to young supplemented group at 1.5 months. Data are presented as mean ± SD, and analysed using ANOVA with Tukey’s *post hoc* analysis. N = 39 (n = 9–10, young control group; n = 9, young supplemented group; n = 5–10, old control group; n = 3–10, old supplemented group).

The effects of TRF supplementation on DNA damage, which is represented by tail length (TL) (Figure 5A), tail DNA percentage (TDP) (Figure 5B), tail moment TM (Figure 5C), and olive moment (OM) (Figure 5D), were measured using the comet assay. In the young control group, tail length, tail moment, and olive moment were significantly reduced after 1.5 months, followed by a significant increase at 3 months. In contrast, the young supplemented group showed a significant increase in tail DNA percentage, tail moment, and olive moment at 3 months compared with baseline and 1.5 months. Interestingly, there was a significant increase in all four parameters in the old supplemented group after 1.5 months of TRF supplementation, which was then significantly reduced after 3 months of supplementation. Notably, at 1.5 months, the old supplemented group showed significantly higher tail length, tail moment, and olive moment than the young supplemented group; however, by 3 months, these levels were significantly lower in the old supplemented group than in the young supplemented group. These findings highlight both age- and time-dependent effects of TRF supplementation on DNA integrity, with older rats demonstrating a beneficial reduction in DNA damage markers following prolonged TRF intake.

### TRF improved muscle regeneration in aged rats

#### Histological findings

**FIGURE 6:**
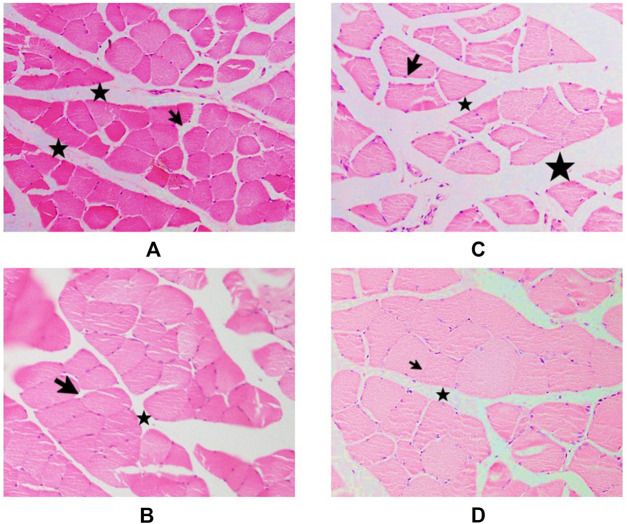
Cross-sectional muscle fibres stained with H&E in rat muscle tissue. Rats supplemented with palm oil: **(A)** young group and **(C)** old group. Rats supplemented with TRF: **(B)** young group, and **(D)** old group. Muscle fibres are surrounded by connective tissue layers (labelled with a star), and each muscle fibre is enveloped by endomysium (labelled with an arrow). The images were taken at ×40 magnification.

**FIGURE 7:**
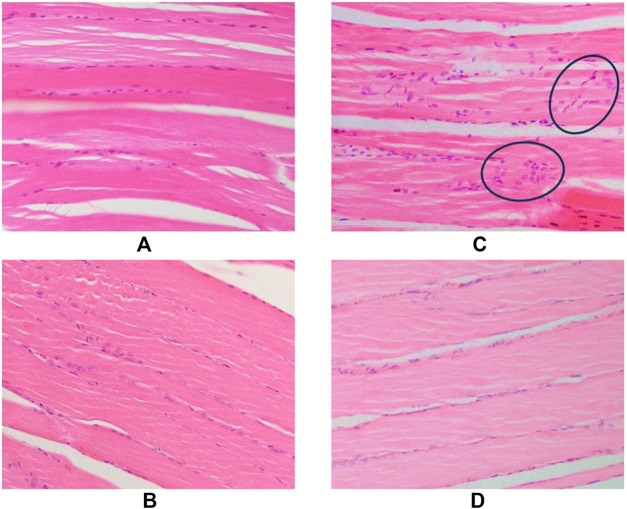
Longitudinal sections of muscle fibres and soleus muscle fascicles. Rats supplemented with palm oil: **(A)** young group, and **(C)** old group. Rat supplemented with TRF: **(B)** young group, and **(D)** old group. Many nuclei can be seen infiltrating into the muscle fibres (circled). The images were taken at ×40 magnification.

Staining of cross-sections of soleus muscle tissue with hematoxylin and eosin (H&E) in control and supplemented groups of young and old rats revealed significant differences in the thickness of connective tissue surrounding the muscle fibres. The connective tissue was thicker in the old control group compared to the young control group (Figures 6A,C), but there was a reduction in thickness in the supplemented groups for both young and old rats (Figures 6B,D). In longitudinal sections of gastrocnemius muscle tissue stained with H&E in control and supplemented groups of young and old rats (Figure 7), it was observed that there were numerous nuclei infiltrating muscle fibres in the old control group (Figure 7C), but not in the young control group (Figure 7A). However, in rats given TRF treatment for 3 months, no nuclear infiltration was observed in either the young or old supplemented groups (Figures 7B,D).

### Correlation between oxidative stress, inflammation, and metabolomics profile

**FIGURE 8:**
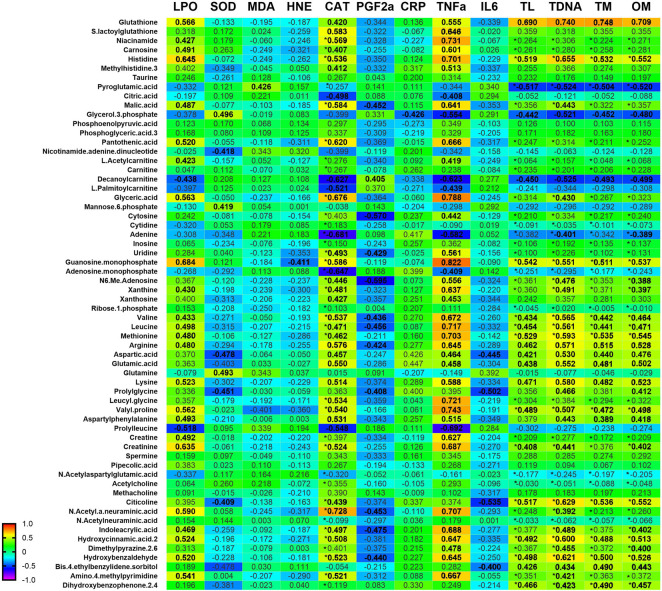
Correlation between oxidative stress markers, inflammatory markers and DNA damage parameters and significant metabolites after 3 months of supplementation in young and old rats (control and supplemented groups). White numbers are Pearson’s correlation, while black numbers are Spearman’s correlation. Bold numbers are considered significant with a p-value <0.05. LPO: Lipid peroxidation; SOD: superoxide dismutase; MDA: Malondialdehyde; HNE: 4-Hydroxynonenal; CAT: Catalase activity; PGF-2α: Prostaglandin Factor 2-alpha; CRP: C-Reactive Protein; TNF-α: Tumour necrosis factor alpha; IL-6: Interleukin-6; TL: Tail Length; TDNA: Tail DNA percentage; TM: Tail Moment; OM: Olive moment.

The heatmap illustrates the correlation patterns among oxidative stress, inflammatory markers, and DNA damage markers with significant metabolites across all groups (Figure 8). Strong positive correlations are observed between metabolites such as glutathione, S-lactoylglutathione, carnosine, citicoline, and leucyl glycine with antioxidant enzymes (CAT, SOD), suggesting potential protective roles. Several metabolites, including taurine, inosine, and leucine, show positive associations with inflammatory markers, whereas certain amino acid derivatives show negative correlations, suggesting possible anti-inflammatory effects. DNA damage parameters (TL, TDP, TM, OM) also correlate positively with metabolites linked to antioxidant activity, where some lipid-derived metabolites exhibit positive associations with DNA damage indices, implying potential pro-damage effects.

**FIGURE 9:**
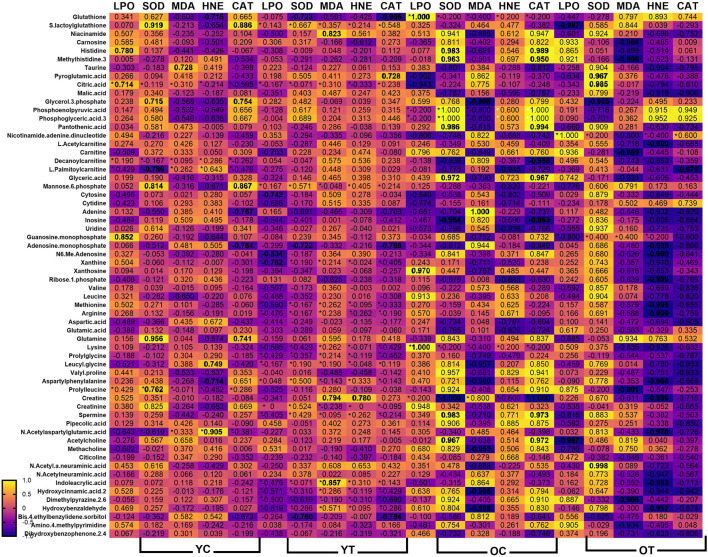
Correlation between oxidative stress markers (LPO, SOD, MDA, HNE, and CAT) and significant metabolites after 3 months of supplementation in young and old rats (control and supplemented groups). White numbers are Pearson’s correlation, while black numbers are Spearman’s correlation. Bold numbers are considered significant with a p-value <0.05. LPO: Lipid peroxidation; SOD: Superoxide dismutase; MDA: Malondialdehyde; HNE: 4-Hydroxynonenal; CAT: Catalase activity; YC: young control; YT: young supplemented; OC: old control; OT: old supplemented.

**FIGURE 10:**
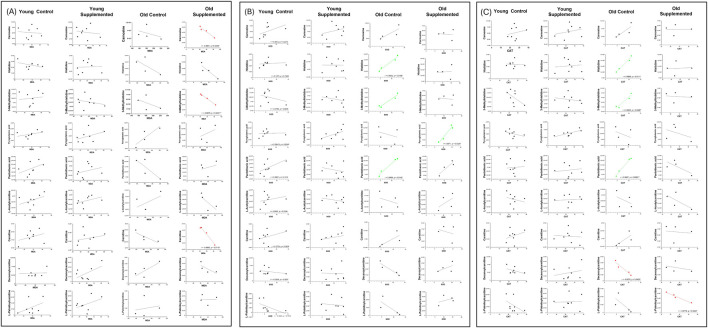
Scatter plots illustrating the correlation between oxidative stress marker (malondialdehyde, MDA) levels and significant metabolites after 3 months of supplementation in young and old rats (control and supplemented groups) **(A)**; Scatter plots illustrating the correlation between antioxidant enzymes superoxide dismutase (SOD), **(B)** and catalase (CAT), **(C)** and significant metabolites after 3 months of supplementation in young and old rats (control and supplemented groups). Each point represents one subject (n = 4 – 8 per group), and correlation values (r) and significance levels (p) are provided on plots, which are significant (red: negatively correlated; green: positively correlated).

Building on the overall correlation patterns observed across all groups, the group-specific heat map further highlights distinct relationships between metabolites and biomarkers influenced by age and supplementation. In the old supplemented (OT) group, metabolites such as carnosine, histidine, 3-methylhistidine, pantothenic acid, L-acetyl carnitine, and carnitine show strong positive correlations with antioxidant enzymes (SOD, CAT), alongside negative correlations with oxidative stress indices (LPO, MDA, HNE) (Figure 9). These trends are substantiated by the scatter plots, where significant negative associations between MDA and carnosine (r = −0.9961, p = 0.0339), 3-methylhistidine (r = −0.9979, p = 0.0021), and carnitine (r = −0.9865, p = 0.0115) in the OT group (Figure 10A). Conversely, for antioxidant parameters, strong positive associations are observed in the old control (OC) groups between SOD activity and histidine (r = 0.9832, p = 0.0168) and pantothenic acid (r = 0.9568, p = 0.0432), and in the OT group between SOD and pyroglutamic acid (r = 0.9617, p = 0.0329) (Figure 10B). Extending these findings, Figure 10C shows a significant correlation between CAT and metabolites in aged animals. In the OC group, CAT activity shows strong positive correlations with histidine, 3-methylhistidine, and pantothenic acid, indicating an age-related coupling between amino acid-associated metabolic pathways and catalase-mediated antioxidant defence. In contrast, these associations are attenuated or absent in the OT group, suggesting that supplementation modulates CAT-linked metabolic responses and may contribute to redox homeostasis. No significant correlations were observed in the young groups. The convergence of findings from the heat map and scatter plots underscores the role of specific metabolites, particularly amino acid derivatives and carnitine species, in modulating oxidative stress and supporting antioxidant capacity, with effects most evident in aged animals receiving supplementation.

**FIGURE 11:**
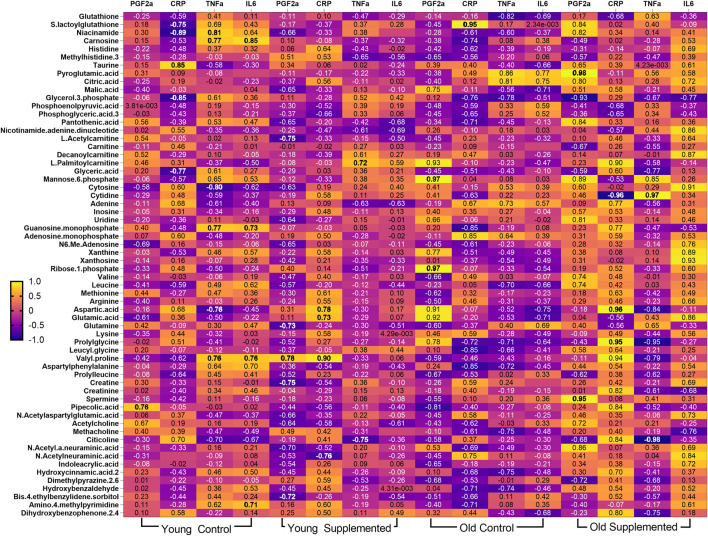
Correlation between inflammatory markers and significant metabolites after 3 months of supplementation in young and old rats (control and supplemented groups). White numbers are Pearson’s correlation, while black numbers are Spearman’s correlation. Bold numbers are considered significant with a p-value <0.05. PGF2α: Prostaglandin factor 2-alpha; CRP: C-Reactive protein; TNF-α: Tumour necrosis factor alpha; IL6: Interleukin 6; YC: young control; YT: young supplemented; OC: old control; OT: old supplemented.

**FIGURE 12:**
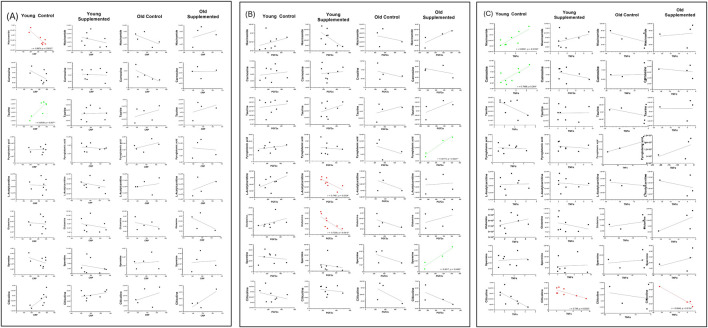
Scatter plots illustrating the correlation between inflammatory marker (C-reactive protein, CRP) and significant metabolites after 3 months of supplementation in young and old rats (control and supplemented groups) **(A)**; Scatter plots illustrating the correlation between prostaglandin factor 2-alpha (PGF-2α), **(B)** and tumor necrosis factor alpha (TNF-α), **(C)** and significant metabolites after 3 months of supplementation in young and old rats (control and supplemented groups). Each point represents one subject (n = 4 – 8 per group), and correlation values (r) and significance levels (p) are provided on plots, which are significant (red: negatively correlated; green: positively correlated).

Extending on the oxidative stress findings, the group-specific heat map of inflammatory markers reveals distinct relationships between metabolites and inflammation, modulated by age and supplementation. In the YC group, niacinamide shows a strong negative correlation with CRP, while taurine exhibits a strong positive correlation (Figure 11). These associations are confirmed in the scatter plots, where CRP is significantly negatively correlated with niacinamide (r = −0.8874, p = 0.0032) and positively correlated with taurine (r = 0.8539, p = 0.0070) (Figure 12A). In the YT group, PGF-2α displays significant negative correlations with L-acetyl carnitine (r = −0.7491, p = 0.0324) and glutamine (r = −0.7258, p = 0.0415), suggesting supplementation-related modulation of inflammatory signalling.

In contrast, the OT group shows a shift towards positive associations, with PGF-2α correlating strongly with pyroglutamic acid (r = 0.9773, p = 0.0227) and spermine (r = 0.9617, p = 0.0483) (Figure 12B). The heat map also suggests that TNF-α shows moderate correlations with several amino acid derivatives and metabolites that are lipid-related across groups, although these did not reach statistical significance in the scatter plots (Figure 12C). Overall, the integration of heat map and scatter plot analysis demonstrates that specific metabolites, particularly niacinamide, taurine, L-acetyl carnitine, glutamine, pyroglutamic acid, and spermine, are strongly associated with inflammatory status, with the direction and strength of these associations varying according to age and supplementation.

**FIGURE 13:**
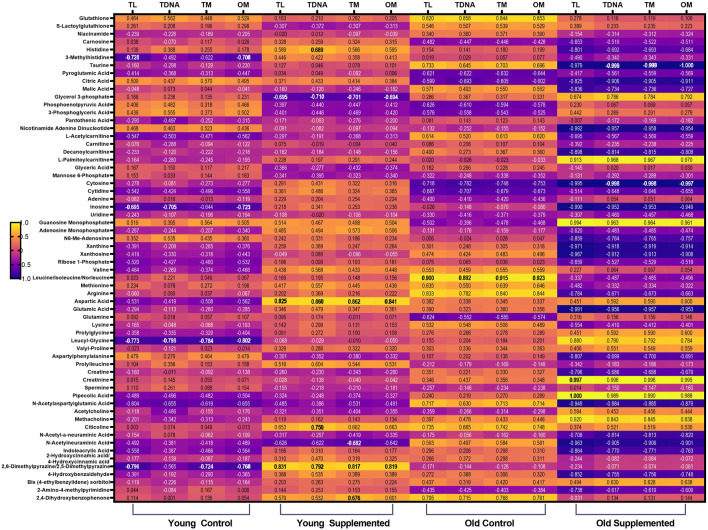
Correlation between DNA damage parameters and significant metabolites after 3 months of supplementation in young and old rats (control and supplemented groups). White numbers are Pearson’s correlation, while black numbers are Spearman’s correlation. Bold numbers are considered significant with a p-value <0.05. TL: tail length; TDNA: tail DNA percentage; TM: tail moment; OM: olive moment; YC: young control; YT: young supplemented; OC: old control; OT: old supplemented.

**FIGURE 14:**
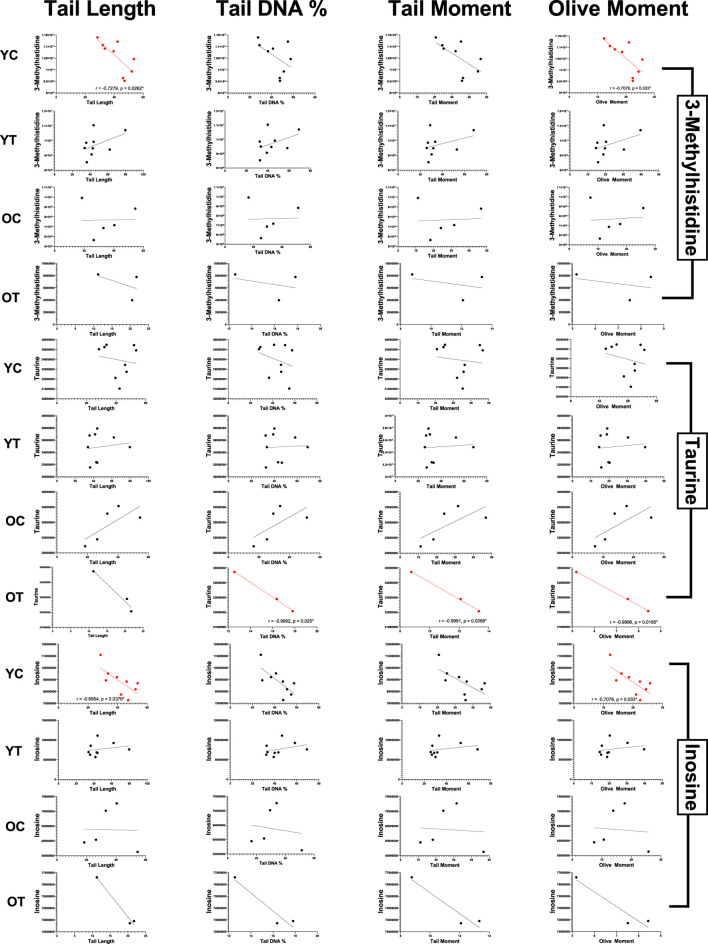
Scatter plots illustrating the correlation between DNA damage indices (tail length, tail DNA %, tail moment, and olive moment) and significant metabolites after 3 months of supplementation in young and old rats (control and supplemented groups). Each point represents one subject (n = 4 – 8 per group), and correlation values (r) and significance levels (p) are provided on plots, which are significant (red: negatively correlated; green: positively correlated). YC, young control; YT; young supplemented; OC, old control and OT, old supplemented.

In relation to the inflammation-associated metabolite patterns, the group-specific heat map for DNA damage parameters (tail length, tail DNA%, tail moment, olive moment) shows clear age- and supplementation-dependent trends (Figure 13). In the YC group, metabolites such as 3-methylhistidine, taurine, and inosine exhibit strong negative correlations with multiple DNA damage indices, suggesting potential protective roles in maintaining genomic stability. These associations are substantiated by the scatter plots, where 3-methylhistidine is significantly negatively correlated with tail length (r = −0.7971, p = 0.0308) and olive moment (r = −0.77876, p = 0.0354), while inosine shows strong negative associations with tail length (r = −0.9304, p = 0.0061) and olive moment (r = −0.9670, p = 0.0017) (Figure 14). In the OT group, taurine stands out with negative correlations to tail DNA% % (r = −0.6902, p = 0.0587) and tail moment (r = −0.6951, p = 0.0569), indicating a possible enhancement of its DNA-protective role through supplementation. The heat map also highlights positive correlations between specific lipid-related metabolites and DNA damage indices in OC rats, although these were not statistically significant. Overall, the data suggest that specific amino acid derivatives and nucleosides, most notably 3-methylhistidine, taurine, and inosine, are associated with reduced DNA damage, with the most potent effects evident in YC and, to a lesser degree, in supplemented aged animals.

## Discussion

Our findings revealed clear and consistent changes in the aged group across various biological parameters, including oxidative stress, inflammation, DNA damage, and metabolite levels. These changes reflect the natural physiological shifts that occur with ageing, particularly the decline in cellular function and redox balance. The significant differences observed between the young and old groups highlight how ageing affects the body at multiple levels, setting the stage for understanding how interventions might help slow or counteract these effects.

One of the key indicators of age-related physiological decline observed in this study was the alteration in body composition. DEXA analysis revealed a significantly higher BMC and BMD in the old groups compared to the young groups, which reflects the natural attainment of peak bone mass in adulthood [40, 41]. Additionally, markers of an increase in fat percentage and fat mass were observed in the older groups, consistent with age-associated fat accumulation reported in previous studies [42, 43]. Notably, a reduction in muscle mass was evident in older animals, indicating early signs of sarcopenia, a progressive loss of muscle tissue commonly associated with ageing.

Although these changes reflect hallmark features of ageing, TRF supplementation did not significantly reverse the decline in muscle mass or overall body composition. This may be due to the advanced age-related deterioration in the study subjects, suggesting that TRF may be more effective as a preventive agent than as a therapeutic intervention. These findings suggest that TRF primarily exerts cytoprotective rather than anabolic effects in ageing muscle. The observed improvements in antioxidant defence, inflammatory modulation, and DNA integrity indicate that TRF supports cellular homeostasis and mitigates molecular damage, rather than directly promoting muscle hypertrophy or reversing established structural decline. The absence of significant effects may also be related to the dosage or duration of TRF administration, suggesting that higher doses or more prolonged supplementation may be necessary to produce measurable improvements. The lack of significant changes in muscle mass may also be influenced by the duration and timing of the intervention. Given that the animals were already aged, structural degeneration may have progressed beyond a reversible stage. It is plausible that earlier or longer-term supplementation could yield more pronounced effects on muscle mass and body composition, particularly if administered prior to the onset of significant age-related deterioration.

The findings in this current study underscore the potential of TRF supplementation to enhance antioxidant defence mechanisms, particularly by improving SOD and CAT activities in aged rats, thereby mitigating age-associated oxidative stress. Ageing can significantly impact SOD and CAT activity and lead to changes in the antioxidant defence system [44]. In fact, the effect of ageing on SOD and CAT activity has been observed in domestic cats, with older cats showing a reduced ability to digest energy and protein, possibly reflecting changes in enzyme activity [44].

The increase in activity of SOD in ageing muscle due to TRF supplementation may be attributed to TRF’s ability to regulate antioxidant enzyme activity. This aligns with previous studies showing a significant increase in SOD activity after 6 months of TRF supplementation [32]. The increase in SOD activity is also consistent with findings that TRF supplementation enhances the proliferative capacity of stress-induced senescent myoblasts and regulates satellite cell renewal, indicating its potential in improving muscle regeneration and function [45]. Furthermore, TRF has been shown to restore the morphology of aged human diploid fibroblasts, thereby reducing senescence-associated β-galactosidase activity and DNA damage, demonstrating its capacity to counteract cellular ageing [46]. Additionally, TRF has been reported to promote myogenic differentiation and prevent replicative senescence of myoblasts, suggesting its potential in preventing muscle ageing [47].

The effects of TRF on catalase activity in muscle during ageing can be explained by considering TRF’s role in regulating antioxidant enzyme activity. Although the references provided do not directly discuss the specific impact of TRF on CAT activity in ageing muscle, a general understanding of TRF’s influence on antioxidant enzymes can provide relevant insights. TRF has been shown to induce the activity of antioxidant enzymes such as SOD [48]. The increased SOD activity due to TRF supplementation may indirectly affect CAT activity, as SOD dismutates superoxide ions to produce hydrogen peroxide, which CAT then decomposes [48]. Furthermore, the therapeutic potential of increased SOD activity in protecting against neurotoxicity caused by oxidative stress may also influence CAT activity [49].

Analysis of MDA and 4-HNE levels revealed a significant elevation in the aged control group relative to the young control group, indicating an age-associated increase in lipid peroxidation and heightened oxidative stress in ageing muscle tissue. Notably, after 3 months of TRF supplementation, the TRF-supplemented aged group exhibited a significant reduction in both MDA and 4-HNE levels compared with the aged control, suggesting a potential antioxidative effect of TRF. This reduction suggests the possible ameliorative effects of TRF on lipid peroxidation and its ability to address age-related oxidative damage. MDA and HNE are markers of lipid peroxidation; thus, their reduction indicates a decline in lipid peroxidation. These findings suggest that even if the overall level of lipid peroxidation does not change significantly, the specific assessment of MDA and HNE provides more detailed and meaningful insights. The observed reduction in MDA and HNE following TRF supplementation highlights TRF’s potential to mitigate lipid peroxidation, underscoring its promise in addressing oxidative stress in ageing muscle tissues.

The increase in MDA and HNE plays a crucial role in muscle ageing through lipid peroxidation. Both of these chemicals, produced from lipid peroxidation, are highly reactive and can form covalent adducts with proteins, indicating increased lipid damage [50]. Previous studies reported elevated plasma levels of MDA and HNE, indicating accelerated lipid peroxidation in various conditions, including uraemia and autoimmune diseases [51]. This mechanism is recognised as a contributing factor in the development of autoimmune conditions and various age-related diseases [52, 53]. The significance of MDA and HNE in lipid peroxidation extends beyond their ability to form protein adducts; they also exert broader toxic and mutagenic effects, highlighting their critical role in mediating oxidative stress [54]. Moreover, elevated levels of MDA and HNE may result from increased hydroxyl radical production, which intensifies oxidative stress and promotes lipid peroxidation [55]. Although overall lipid peroxidation levels did not change significantly after TRF supplementation in the aged group, the significant reductions in MDA and HNE suggest that TRF may mitigate lipid peroxidation in the context of muscle ageing. These findings offer a more detailed and meaningful perspective on the effects of TRF on lipid peroxidation in ageing muscle tissue.

TRF supplementation has been shown to reduce MDA and HNE values after 3 months of supplementation. This may be due to TRF’s potent antioxidant properties, which can scavenge free radicals and ROS generated during lipid peroxidation. By neutralising these harmful molecules, TRF may help prevent the formation of MDA and 4HNE, thereby reducing oxidative stress levels [56]. Moreover, previous studies have confirmed that tocotrienols can inhibit lipid peroxidation by preventing lipid oxidation in cell membranes, thus reducing the availability of substrates for MDA and 4HNE formation [57]. Studies have also shown that tocotrienols can modulate the activity of enzymes involved in lipid metabolism and antioxidant defence pathways [58]. In the present study, TRF was found to enhance the activity of antioxidant enzymes such as SOD, which plays a crucial role in neutralising ROS and mitigating lipid peroxidation. This finding aligns with previous studies indicating that SOD catalyses the dismutation of superoxide radicals into oxygen and hydrogen peroxide, thereby lowering superoxide radical concentration, which is a major contributor to ROS and lipid peroxidation [59]. By reducing superoxide levels, SOD helps prevent the initiation of lipid peroxidation, a chain reaction that produces lipid hydroperoxides, which subsequently form MDA and 4HNE [60].

Additionally, TRF has been proven to possess anti-inflammatory properties that can reduce inflammation caused by oxidative stress [61]. By reducing inflammatory responses, TRF may indirectly lower MDA and 4-HNE production, which are often associated with inflammatory processes. In this study, the effects of TRF supplementation on various inflammatory markers were investigated across different age groups. Specifically, the levels of CRP, IL6, TNF-α, and PGF-2α were measured in control and TRF-supplemented groups. In the control group, CRP levels were significantly higher compared to the supplemented group. Moreover, IL6 and TNF-α levels decreased in the young control group after 3 months of observation, although no other significant changes were observed. Interestingly, PGF-2α levels were significantly higher in the old supplemented group after 1.5 months of receiving TRF. These findings suggest that TRF supplementation may have potential anti-inflammatory effects, as indicated by the observed reduction in CRP levels and modulation of IL6 and TNF-α in the young control group.

The findings on increased CRP levels are consistent with previous studies recognising CRP as a marker of systemic inflammation associated with age-related muscle deterioration, known as sarcopenia [62]. Studies also report that increased inflammatory signalling in ageing muscle may reduce muscle regeneration potential, further contributing to muscle ageing [63]. Moreover, previous studies indicated that elevated CRP levels in the context of muscle ageing may reflect a state of chronic low-grade inflammation associated with structural and functional changes in aged muscle [64]. Additionally, high levels of pro-inflammatory cytokines, including CRP, IL6, and TNF-α, have been linked to muscle wasting and increased muscle ageing. This suggests their involvement in complex inflammatory signalling networks related to muscle homeostasis and age-associated impairments [65].

However, the measured IL6 and TNF-α levels did not show a significant increase, as expected, given prior studies showing that pro-inflammatory markers such as IL6 and TNF-α increase with age. The lack of increase or detection of IL6 and TNF-α levels in this study after 3 months may be due to timing mismatches with the peak expression of these cytokines, which may have transiently increased earlier or later than the time of measurement. Another reason could be that the supplementation or intervention successfully suppressed IL6 and TNF-α production. Another possible reason for the lack of increase in IL6 and TNF-α levels is the inevitable variability in cytokine levels among individual rats. Factors such as genetics, lifestyle, and overall health status can influence cytokine production. Furthermore, inflammation is a complex and dynamic process involving multiple cytokines and signalling pathways. Although IL6 and TNF-α are key players, other cytokines and mediators may also be involved in the inflammatory responses. Changes in IL6 and TNF-α may be masked by compensatory mechanisms or interactions with other inflammatory molecules.

In the current study, PGF-2α was measured via a urine test. Urinary PGF-2α may serve as an inflammatory marker in ageing muscle. In older and obese individuals, higher levels of circulating inflammatory markers and/or cytokines are associated with low muscle content, suggesting that muscle tissue itself may regulate inflammatory markers [66]. PGF-2α is a bioactive prostaglandin associated with various physiological and pathological processes, including inflammation [67]. Prior research has demonstrated that 8-iso-PGF2α, a type of isoprostane, can suppress monocyte adhesion to microvascular endothelial cells, which is an initial step in the inflammatory response. This suggests its role in modulating inflammation [68].

Oxidative stress, characterised by an imbalance between ROS generation and the body’s antioxidant defence capacity, can lead to damage of cellular components, including DNA [69, 70]. The buildup of oxidative DNA damage, exemplified by the formation of 8-hydroxy-2′-deoxyguanosine (8-OHdG), has been documented during ageing and in numerous pathological conditions, highlighting the contribution of oxidative stress to DNA damage [71]. Additionally, chronic inflammation has also been linked to ROS production, which can directly contribute to DNA damage and genomic instability [70]. The roles of DNA damage in age-associated inflammation are also well-documented, where excess cytoplasmic DNA is thought to drive inflammatory responses and cellular senescence [71]. Together, oxidative stress, DNA damage, and inflammation form an interconnected network that contributes to ageing and age-related diseases.

Earlier studies have reported a progressive increase in the percentage of DNA present in the comet tail with advancing age, indicating a gradual accumulation of DNA damage over time [72]. These observations are in line with the findings of the present study, which demonstrated increased DNA damage in aged rats.

Histological analysis complemented these findings by revealing a consistent reduction in connective tissue thickness and nuclear infiltration in the muscle of TRF-supplemented rats. This provides strong evidence for TRF’s potential to enhance muscle function and counteract age-related changes in body composition. In the context of metabolomic analysis, TRF supplementation led to significant shifts in muscle metabolite profiles associated with tissue remodelling, fiber atrophy, and mitochondrial function in both young and old rats.

In this study, metabolite changes were compared between the control and TRF-supplemented groups in young and old rats using muscle tissue. The metabolomic findings, derived from the same experimental cohort, were previously reported in an earlier publication from this study [39]. In this present article, we extended this analysis by performing correlation analysis between the metabolomic data and the physiological and biochemical parameters, providing deeper insight into the functional relevance of the observed metabolic shifts. The observed correlations suggest that TRF appears to modulate redox-related amino acid and acylcarnitine metabolism in aged muscle in a manner indicative of reduced oxidative damage and refined regulation of antioxidant enzyme activity, rather than a simplistic linear relationship between antioxidant availability and enzymatic response. This is supported by the correlations between metabolites and biomarkers observed, which collectively suggest a shift toward improved endogenous antioxidant buffering and mitochondrial metabolic regulation.

Key metabolites implicated in redox homeostasis, particularly carnosine and histidine-related compounds, demonstrated strong associations with oxidative stress markers. Carnosine, a histidine-containing dipeptide abundant in skeletal muscle, is known to scavenge reactive oxygen and nitrogen species, chelate redox-active metals, and form adducts with lipid peroxidation products while supporting glutathione (GSH) homeostasis [73, 74]. Its negative correlation with MDA in the TRF-supplemented group suggests that higher carnosine availability is linked to reduced lipid peroxidation, consistent with its established antioxidant and anti-glycation roles [75]. Similarly, histidine and its derivatives, which contribute to intracellular buffering and metal ion interaction [76], showed positive correlations with SOD and catalase in the control group, indicating that under higher oxidative stress conditions, histidine metabolism may track with increased enzymatic antioxidant demand [77].

In contrast, 3-methylhistidine, a marker of myofibrillar proteolysis [78], exhibited positive correlations with SOD and catalase in older controls, reflecting a state in which muscle protein breakdown, oxidative stress, and compensatory antioxidant enzyme activity are closely coupled [79, 80]. This pattern is characteristic of sarcopenic muscle, where chronic oxidative stress drives both proteolysis and upregulation of antioxidant defences [81]. Similarly, pantothenic acid, a precursor of coenzyme A essential for mitochondrial β-oxidation [82], was positively associated with antioxidant enzymes in controls, suggesting adaptive upregulation of energy metabolism and redox defences in response to metabolic stress [83].

TRF supplementation altered these relationships substantially. Negative correlations between carnosine, 3-methylhistidine, and carnitine with MDA, alongside inverse relationships between long-chain acylcarnitines and catalase activity, indicate a metabolic environment characterized by lower oxidative burden and reduced reliance on maximal antioxidant enzyme induction [75]. The emergence of a positive association between pyroglutamic acid, an intermediate of the γ-glutamyl cycle [84, 85], SOD further suggests tighter coupling between glutathione cycling and superoxide detoxification under TRF [86]. This may reflect a more efficient or regulated redox buffering system, where low-molecular-weight antioxidants and glutathione metabolism play a more prominent role relative to stress-driven enzymatic responses.

Importantly, the divergence in correlation patterns between control and TRF-supplemented groups suggests a shift in how antioxidant systems integrate metabolic signals. In the control state, positive associations between histidine, 3-methylhistidine, pantothenic acid, and antioxidant enzymes are consistent with a response driven by damage, wherein increased oxidative stress simultaneously promotes muscle breakdown, metabolic adaptation, and enzyme upregulation [87]. In contrast, the TRF-associated pattern, which is characterized by reduced lipid peroxidation, altered acylcarnitine handling, and diminished coupling between proteolysis markers and antioxidant enzymes, supports a model in which primary oxidative stress is attenuated, thereby reducing the need for sustained high enzymatic antioxidant activity [29].

These findings align with established features of sarcopenia, including elevated oxidative stress, increased lipid peroxidation, mitochondrial dysfunction, and enhanced myofibrillar proteolysis [88]. The observed TRF-associated metabolomic shifts, particularly the enhanced role of carnosine, glutathione cycle intermediates, and carnitine metabolism, are consistent with improved mitochondrial redox handling and attenuation of oxidative damage [29, 39]. While correlation analysis does not establish causality, these data, together with prior mechanistic evidence demonstrating TRF-mediated modulation of antioxidant enzyme expression and lipid peroxidation, support the interpretation that TRF contributes to a partial normalization of the redox-metabolic milieu in ageing muscle. This normalization appears to involved a transition from stress-responsive, enzyme-dominated antioxidant activity toward a more balances system integrating intrinsic antioxidant metabolites and mitochondrial metabolic flexibility.

To gain deeper insight into the metabolic pathways linked to oxidative stress, inflammation, and DNA damage in ageing, we conducted Pearson’s correlation analysis between the identified metabolites and relevant biochemical markers. This strategy enables us to pinpoint metabolites that may serve as indicators or regulators of these physiological changes.

Findings from the same cohort study, previously published [39] reported significant alterations in a range of metabolites in the aged control group compared to the young control group. These included notable changes in amino acids, dipeptides, nucleosides, and metabolic intermediates such as histidine, 3-methylhistidine, hydroxycinnamic acid, phenylalanine, leucyl-glycine, valyl-proline, prolyleucine, aspartyl phenylalanine, N6-Me-adenosine, decanoyl carnitine, L-palmitoyl carnitine, glyceric acid, malic acid, xanthine, tyrosine, tryptophan, pantothenic acid, and uridine [39]. These shifts reflect age-associated metabolic dysregulation and provide a biochemical basis for further investigation into oxidative stress, inflammation, and genomic instability observed with ageing.

The correlation analysis between selected metabolites and oxidative stress markers revealed distinct age- and supplement-dependent metabolic responses in skeletal muscle. In aged control rats, a strong positive correlation between CAT and the metabolites histidine, pantothenic acid, and 3-methylhistidine points to the body’s adaptive antioxidant response to ageing-related oxidative stress [89]. Histidine, known for its free radical-scavenging and buffering properties, along with its derivative anserine (1-methylhistidine), supports mitochondrial and redox balance during oxidative challenge [90]. Pantothenic acid, a precursor of coenzyme A (CoA), plays a key role in energy metabolism and redox regulation [91, 92]. Its adequacy is vital for mitochondrial function, and elevated levels have been linked to increased antioxidant activity, including glutathione synthesis, which suggests a protective metabolic adjustment during oxidative stress [92, 93]. Additionally, higher levels of 3-methylhistidine, which is an indicator of muscle protein breakdown, may reflect increased muscle turnover to support energy or antioxidant demands during ageing, underlining the importance of maintaining muscle health and nutrient intake in the elderly [94, 95].

TRF supplementation appears to moderate the association between CAT and metabolites like histidine and pantothenic acid in aged rats, suggesting improved redox balance and reduced oxidative stress. This shift implied a lowered physiological demand for compensatory antioxidant responses. In the control group, strong CAT and metabolite correlations indicate a heightened need for oxidative stress management. In contrast, the weakened correlation seen with TRF supplementation suggests stabilised metabolic and redox states, likely due to enhanced mitochondrial and cellular function [96, 97]. Furthermore, TRF may modulate gene expression linked to metabolic stress and inflammation, supporting muscle recovery and resilience in ageing populations through its impact on amino acid pathways, including those involving histidine [97, 98].

In aged controls, a strong positive correlation between SOD and the selected metabolites highlights the importance of glutathione metabolism and CoA synthesis in managing oxidative stress. Histidine contributes to the production of antioxidant peptides like anserine and carnosine, while pyroglutamic acid supports glutathione activity [99, 100]. These relationships suggest a coordinated antioxidant response in ageing. However, ageing is often marked by reduced antioxidant enzyme activity, increasing the need for compensatory mechanisms [101]. Studies show that supplementation with TRF boosts SOD and glutathione activity, reducing oxidative burden and improving redox balance [32, 97]. The weakened correlations between SOD and these metabolites after TRF supplementation suggest a reduced need for metabolic compensation, reflecting enhanced antioxidant capacity. TRF likely exerts its effects by upregulating SOD and supporting mitochondrial health, possibly via Nrf2 pathway activation, which improves the body’s natural antioxidant defence [102].

In TRF-supplemented aged rats, the negative correlation between MDA and metabolites like carnosine and carnitine suggests TRF’s strong antioxidant potential in reducing oxidative damage. Since high MDA reflects oxidative stress-related cellular damage, its reduction suggests that TRF supports a better redox balance in ageing muscle [103]. Carnosine, known for buffering and antioxidant functions, helps neutralise ROS and protect muscle cells [104]. Carnitine aids in mitochondrial fatty acid transport, promoting energy metabolism and muscle function [105]. Their inverse relationship with MDA suggests that TRF plays a role in preserving these metabolites, thereby enhancing cellular defence and muscle health. Studies confirm TRF’s ability to lower oxidative markers like MDA and improve energy metabolism by boosting antioxidant enzymes [96, 106]. This not only protects muscle tissue but may also combat age-related sarcopenia and frailty by maintaining key metabolites and reducing oxidative burden [107].

The significant negative correlations between PGF2α, an inflammatory mediator, and metabolites such as glutamine and L-acetyl carnitine in young rats supplemented with TRF suggest that TRF plays a key role in regulating inflammation, supporting metabolic balance under stress. PGF2α is known to rise during oxidative stress or tissue injury, and its inverse relationship with glutamine indicates a potential compensatory increase in this amino acid to support antioxidant defence, glutathione synthesis, and tissue repair [108, 109]. Similarly, the inverse relation with L-acetyl carnitine, a metabolite involved in fatty acid oxidation and mitochondrial function, suggests that TRF may enhance cellular energy metabolism while reducing inflammation [110]. L-acetyl carnitine has been shown to suppress pro-inflammatory cytokines and improve mitochondrial bioenergetics, contributing to a more efficient and adaptive response to oxidative and inflammatory stress [111, 112]. These findings indicate that TRF supplementation supports mitochondrial health and amino acid metabolism, creating a protective mechanism that reduces PGF2α levels and helps maintain cellular function under physiological stress.

The strong inverse correlation between citicoline and TNF-α in old rats suggests that TRF may reduce inflammation by enhancing membrane integrity and suppressing pro-inflammatory cytokines. Citicoline, a precursor of phosphatidylcholine, supports membrane stability and has documented anti-inflammatory and neuroprotective effects, which are particularly beneficial in ageing tissues prone to oxidative and inflammatory damage [113, 114]. Conversely, the positive correlations between PGF2α, spermine, and pyroglutamic acid reflect heightened metabolic activity in response to inflammation. Spermine, a polyamine involved in cell maintenance and stress responses, appears to increase alongside inflammation, suggesting a compensatory signalling mechanism in aged muscle [115]. Pyroglutamic acid, linked to glutathione metabolism, likely rises to support antioxidant defence under inflammatory stress, indicating an adaptive attempt to maintain redox balance [116]. In contrast, aged control rats exhibited weak or disrupted correlations between inflammatory markers and metabolic indicators, suggesting impaired metabolic responsiveness and flexibility. This breakdown in metabolic-inflammation coupling is consistent with chronic inflammation in ageing and highlights TRF’s role in restoring more adaptive biochemical responses [117, 118].

In young control rats, positive correlations between 3-methylhistidine and tail length and olive moment suggest that muscle protein breakdown in youth may reflect active regeneration and healthy muscle function rather than damage or decline [119, 120]. This indicates a more resilient and dynamic metabolic state in younger animals. However, this association weakens in aged or TRF-supplemented rats, likely due to age-related metabolic changes such as reduced muscle mass, hormonal shifts, and impaired protein synthesis [121, 122]. Although TRF offers antioxidant benefits, it may not fully counteract the diminished metabolic adaptability seen with ageing [47, 123]. The lack of this protective correlation in older or TRF-supplemented animals highlights the complexity of age-related metabolic regulation. It suggests that while 3-methylhistidine is a useful marker of muscle catabolism, its interpretation should consider the broader physiological context, particularly age and oxidative stress levels [94, 122].

The correlations between taurine levels and DNA fragmentation markers in old supplemented rats highlight taurine’s potential role in protecting against age-related oxidative stress and DNA damage. Negative associations with tail DNA% %, tail moment, and olive moment suggest that higher taurine levels are linked to lower DNA damage in aged muscle tissue [124, 125]. Taurine is known for its antioxidant and membrane-stabilising properties, which help reduce oxidative stress and support cellular health. Importantly, taurine is highly enriched in skeletal muscle, where it regulates ion channel function, maintains membrane stability, and supports mitochondrial quality control and calcium homeostasis, while also acting as a key cytoprotective osmolyte and indirect antioxidant. Consistent with these roles, previous studies have demonstrated that taurine supplementation improves oxidative balance, enhances mitochondrial function, and attenuates muscle atrophy, partly through modulation of key signalling pathways such as P13K/Akt/mTOR and NF-κB/FOXO-Atrogin1/MuRF1 that are central to muscle proteostasis [126]. In conditions of oxidative stress or muscle overuse, taurine has also been shown to reverse oxidative damage and restore contractile function, which may contribute to limiting DNA damage [127], in line with the present findings.

Interestingly, these protective correlations were absent in both young and old control groups, indicating that TRF may enhance taurine’s antioxidant effects specifically in older animals under oxidative stress [124]. This suggests that TRF is essential in amplifying taurine’s protective role against DNA damage in aged tissues. The synergy between taurine and TRF supports interpreting taurine not merely as a passive biomarker, but as part of an adaptive antioxidant and membrane-stabilising response that becomes more evident when oxidative stress is pharmacologically attenuated. Collectively, this interaction represents a promising therapeutic strategy to reduce age-related muscle degeneration and genomic instability [128]. As ageing is marked by decreased repair capacity and increased oxidative damage, this combination may help preserve muscle integrity and counteract frailty in older adults [129].

The negative correlations between inosine levels and markers of DNA damage in young control rats suggest that inosine may play a protective role in preserving DNA integrity during early life. As a purine nucleoside with anti-inflammatory and cytoprotective properties, higher inosine levels appear to be linked with reduced DNA fragmentation, indicating a supportive role in maintaining healthy muscle tissue when DNA repair processes are more active [130]. Inosine is known to combat oxidative stress and has shown protective effects in various cell models [131]. Its effectiveness seems more pronounced during youth, a period marked by cellular repair capacity and high metabolic activity. However, the lack of similar associations in older or supplemented animals suggests that ageing or interventions may diminish inosine’s ability to activate these protective pathways, reflecting shifts in metabolic and repair mechanisms over time [132].

## Summary table

### What is known about this topic

- Ageing increases oxidative stress, inflammation, and DNA damage, contributing to sarcopenia.
- Metabolic dysregulation is closely linked to redox imbalance and muscle degeneration.
- Tocotrienols possess antioxidant and anti-inflammatory properties with potential protective effects.

### What this work adds

- Demonstrates integrated correlations between metabolomics, oxidative stress, inflammation, and DNA damage in ageing muscle.
- Shows TRF enhances antioxidant defence and modulates metabolite-biomarker interactions in aged rats.
- Identifies specific metabolites (e.g., taurine, carnitine, histidine) linked to improved redox and genomic stability.

This work represents an advance in biomedical science by linking metabolomic shifts to redox, inflammatory, and genomic changes, demonstrating TRF’s role in stabilising these interactions during ageing.

Beyond individual biomarker changes, ageing is increasingly recognised as a process characterised by dysregulation of interconnected metabolic and redox networks [133]. The findings of the present study support this systems-level perspective, demonstrating that ageing is associated with a coordinated, yet maladaptive coupling between oxidative stress, inflammation, and metabolic pathways [134]. Importantly, TRF supplementation appears to stabilise these interactions, shifting the system from a predominantly “damage-drive” state toward a more regulated and resilient metabolic environment. This transition is reflected in the attenuation of pathological metabolite-biomarker associations and the enhancement of protective metabolic linkages. Collectively, these findings extend the understanding of TRF beyond its conventional role as a direct antioxidant, highlighting the potential to modulate the broader redox-metabolic network underlying ageing and sarcopenia.

## Conclusion

In conclusion, this study demonstrates that ageing is associated with significant physiological, biochemical, and metabolic alterations, including increased oxidative stress, inflammation, DNA damage, and disrupted metabolite profiles. Although TRF supplementation did not significantly restore muscle mass or overall body composition, it effectively enhanced antioxidant defence by increasing SOD and CAT activities, reducing lipid peroxidation (MDA, 4-HNE), and modulating key metabolites associated with redox balance, inflammation, and DNA damage. This study utilised only male rats; therefore, the findings may not fully capture sex-specific differences in redox regulation, metabolism, and response to TRF supplementation. Further studies incorporating both sexes are warranted to determine whether the observed metabolite-biomarker interactions and TRF effects are sex-dependent.

Notably, TRF influences metabolite-biomarker interactions, including associations involving protective metabolites such as taurine, and contributes to the attenuation of inflammatory markers such as CRP and PGF-2α. These findings suggest that TRF primarily modulates redox and inflammatory pathways and supports metabolic homeostasis in ageing muscle, rather than reversing established structural or compositional decline.

**FIGURE 15:**
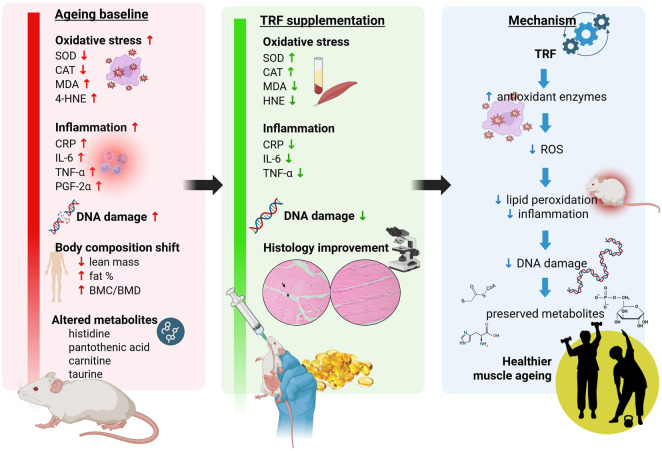
Graphical summary of the effects of tocotrienol-rich fraction (TRF) supplementation on ageing skeletal muscle. Ageing was associated with increased oxidative stress, inflammation, DNA damage, altered body composition, and metabolic dysregulation. TRF supplementation enhanced antioxidant enzyme activity (SOD, CAT), reduced lipid peroxidation (MDA, 4-HNE) and inflammatory markers (CRP, IL-6, TNF-α, PGF-2α), attenuated DNA fragmentation, improved histological features, and restored metabolite balance. Collectively, these effects support improved redox homeostasis, genomic stability, and healthier muscle ageing (Created with Biorender.com).

Overall, the correlation analyses indicate that ageing is characterised by coordinated metabolic shifts linking amino acid and carnitine metabolism with antioxidant defense, inflammation, and genomic stability, while TRF supplementation partially mitigates these age-associated alterations. Collectively, these findings support the potential role of TRF as a nutritional strategy to preserve redox balance and cellular integrity during ageing. A schematic summary of these interactions is presented in Figure 15.

### Limitations and future studies

One notable limitation of this study is the relatively brief duration of TRF supplementation, which may have been insufficient to produce substantial improvements in muscle mass or fully counteract age-related degenerative changes. Future research should explore longer intervention periods to better understand TRF’s therapeutic potential. In addition, blinding was not implemented during group allocation and outcome assessment; however, standardised experimental protocols were consistently applied to minimize potential bias. Future studies incorporating blinded designs would further strengthen methodological rigor.

Furthermore, this study did not include functional assessments of muscle performance, such as grip strength or endurance capacity, which could complement the biochemical and histological findings. Nevertheless, the present study provides robust mechanistic insights into the effects of TRF on oxidative stress, inflammation, DNA integrity, and metabolic regulation in ageing muscles. Further investigations integrating functional outcomes alongside molecular and structural analyses would further enhance the translational relevance of these findings.

## Data Availability

The original contributions presented in the study are included in the article/Supplementary Material, further inquiries can be directed to the corresponding author.
