# Supplementary figures and images for: Correlation between oxidative stress and inflammation with metabolomics profile in skeletal muscle of ageing animal model and its modulation by tocotrienol-rich fraction
Source: Br J Biomed Sci. 2026 May 8;83:16208. doi: 10.3389/bjbs.2026.16208 (PMC13194080; doi:10.3389/bjbs.2026.16208)

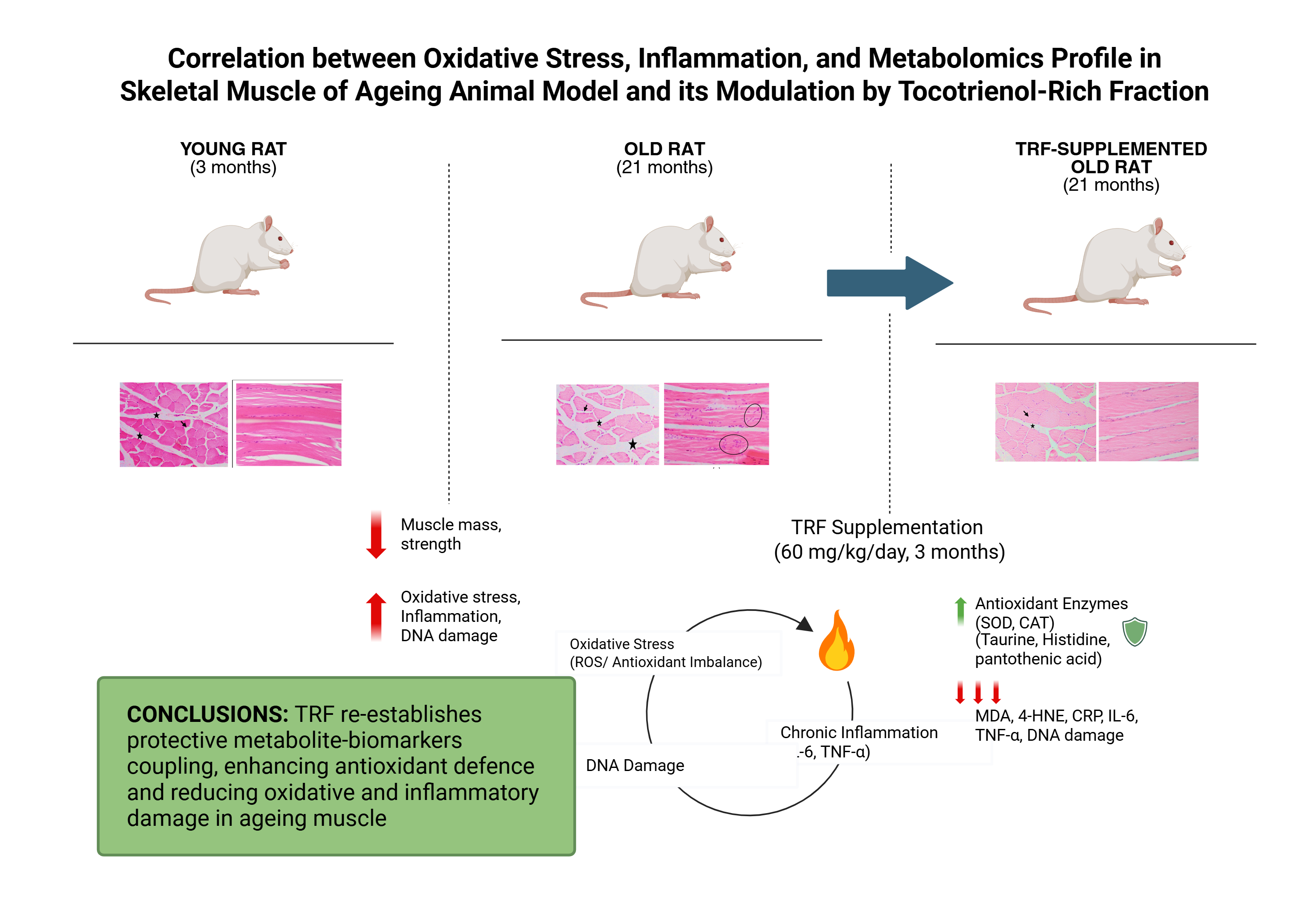

Supplement: Supplementary file 1 [file Image1.jpeg]
